# A character theoretic formula for the base size

**DOI:** 10.1007/s00013-025-02120-2

**Published:** 2025-03-24

**Authors:** Coen del Valle

**Affiliations:** School of Mathematics and Statistics, University of St Andrews, St Andrews, UK

**Keywords:** Base size, Irreducible character, Large base, 20B05, 20B30

## Abstract

A base for a permutation group *G* acting on a set $\Omega$ is a sequence ${\mathcal {B}}$ of points of $\Omega$ such that the pointwise stabiliser $G_{{\mathcal {B}}}$ is trivial. The base size of *G* is the size of a smallest base for *G*. We derive a character theoretic formula for the base size of a class of groups admitting a certain kind of irreducible character. Moreover, we prove a formula for enumerating the non-equivalent bases for *G* of size $l\in {\mathbb {N}}.$ As a consequence of our results, we present a very short, entirely algebraic proof of the formula of Mecenero and Spiga for the base size of the symmetric group $\textrm{S}_n$ acting on the *k*-element subsets of $\{1,2,3,\ldots ,n\}.$ Our methods also provide a formula for the base size of many product type permutation groups.

## Introduction

A *base* for a permutation group *G* acting on a finite set $\Omega$ is a sequence ${\mathcal {B}}$ of points of $\Omega$ with trivial pointwise stabiliser $G_{\mathcal {B}}.$ The size *b*(*G*) of a smallest base for *G* is called the *base size* of *G*. In 1992, Blaha [2] showed that the problem of finding a minimum base for an arbitrary group *G* is NP-hard. Despite this, much work has been done towards determining the base size of certain families of groups, especially primitive groups.

Let *n* and *k* be positive integers with $n>2k$ and let $\textrm{S}_{n,k}$ be the symmetric group $\textrm{S}_n$ acting on the *k*-element subsets of $[n]:=\{1,2,\ldots ,n\}.$ Recently, in two independent papers [3, 4], precise formulae were determined for the base size $b(\textrm{S}_{n,k}),$ both of which take remarkably different forms. The two results are proved using predominantly combinatorial techniques, the arguments mostly using the language of graphs and hypergraphs. During the review process, an anonymous referee made an astute observation: the beautiful formula of Mecenero and Spiga [4, Theorem 1.1] has an entirely character theoretic interpretation. In particular, after some straightforward algebraic manipulation, one derives that if ${{\textrm{sgn}}}$ is the sign character of $\textrm{S}_n$ and $\chi$ is the permutation character of $\textrm{S}_{n,k},$ then

$$\begin{aligned} b(\textrm{S}_{n,k})=\min \{l\in {\mathbb {N}}: \langle {{\textrm{sgn}}},\chi ^l\rangle \ne 0\}. \end{aligned}$$

Recently, Pablo Spiga asked whether this connection was purely coincidental. This paper gives an entirely character theoretic formula for the base size of groups admitting a certain kind of homomorphism which we call *base-controlling*.

Let $\Omega$ be a finite set, and let $G\le {\textrm{Sym}}(\Omega ).$ We say that a homomorphism ${\phi :G\rightarrow \{1,-1\}}$ is *base-controlling* if for every tuple ${\mathcal {A}}$ of points of $\Omega ,$ ${\mathcal {A}}$ is a base if and only if $\phi (G_{{\mathcal {A}}})=1.$ Note that any base-controlling homomorphism of *G* is an irreducible character of *G*. Define $\langle -, - \rangle$ to be the standard scalar product of (complex-valued) class functions. That is, for class functions $\varphi _1,\varphi _2$ of *G*, 

$$\begin{aligned} \langle \varphi _1,\varphi _2\rangle =|G|^{-1}\sum _{g\in G}\varphi _1(g)\overline{\varphi _2(g)}. \end{aligned}$$

Our first main result allows us to enumerate the non-equivalent bases of any given length. A *G*-orbit ${\mathcal {O}}$ is called *regular* if $G_x=1$ for $x\in {\mathcal {O}}.$ A base of size *l* for *G* acting on $\Omega$ is thus a point of a regular orbit of *G* acting on $\Omega ^l.$

### Theorem 1.1

Let *G* be a permutation group acting on a set $\Omega$ with permutation character $\chi .$ If *G* admits a base-controlling homomorphism $\phi ,$ then *G* has exactly $\langle \phi ,\chi ^l\rangle$ regular orbits on $\Omega ^l.$

As an immediate consequence of Theorem 1.1, we deduce a character theoretic formula for the base size, generalising [4, Theorem 3.1].

### Theorem 1.2

Let *G* be a permutation group with permutation character $\chi .$ If *G* admits a base-controlling homomorphism $\phi ,$ then

$$\begin{aligned} b(G)=\min \{l \in {\mathbb {N}}: \langle \phi ,\chi ^l\rangle \ne 0\}. \end{aligned}$$

Theorem 1.1 has another powerful consequence. Let *G* be as in the set-up of Theorem 1.1, and let $P\le \textrm{S}_m$ for some *m*. Define *D*(*P*) to be the *distinguishing number* of *P*,  that is, the minimum number of parts in a partition of [*m*] such that the pointwise stabiliser in *P* of these parts is trivial. In [1], it is shown that $b(G\wr P)$ (with product action) is the minimum *l* such that the number of regular orbits of *G* on $\Omega ^l$ is at least *D*(*P*),  hence we deduce the following corollary to Theorem 1.1.

### Theorem 1.3

Let *G* be a permutation group with permutation character $\chi ,$ and let $P\le \textrm{S}_m$ for some integer *m*. If *G* admits a base-controlling homomorphism $\phi ,$ then

$$\begin{aligned} b(G\wr P)=\min \{l\in {\mathbb {N}}: \langle \phi , \chi ^l\rangle \ge D(P)\}. \end{aligned}$$

In Section 2, we prove Theorem 1.1, hence deducing Theorems 1.2 and 1.3. In Section 3, we discuss some examples and non-examples of groups admitting base-controlling homomorphisms, in particular, we recover the formula for $b(\textrm{S}_{n,k})$ of Mecenero and Spiga [4], and deduce sharp bounds on the base size of large base groups.

## Proving Theorem 1.1

Throughout this section, let *G* be a permutation group acting on a finite set $\Omega$ with permutation character $\chi .$ Suppose that *G* admits a base-controlling homomorphism $\phi ,$ and define $K:=\ker \phi .$ Given $l\in {\mathbb {N}},$ let *o*(*l*) and $o_K(l)$ be the numbers of *G*-orbits and *K*-orbits on $\Omega ^l,$ respectively.

### Lemma 2.1

Fix $$l\in {\mathbb {N}}.$$ Then $$\langle \phi ,\chi ^l\rangle =o_K(l)-o(l).$$

### Proof

By the orbit-counting lemma,

$$\begin{aligned} o(l)=|G|^{-1}\sum _{g\in G}\textrm{fix}_{\Omega ^l}(g)=|G|^{-1}\left( \sum _{g\in K}\chi (g)^l\right) +|G|^{-1}\left( \sum _{g\in G\setminus K}\chi (g)^l\right) , \end{aligned}$$

whilst

$$\begin{aligned} o_K(l)=|K|^{-1}\left( \sum _{g\in K}\chi (g)^l\right) =2\cdot |G|^{-1}\left( \sum _{g\in K}\chi (g)^l\right) . \end{aligned}$$

Therefore,

$$\begin{aligned} o_K(l)-o(l)= &   \left( |G|^{-1}\sum _{g\in K}\chi (g)^l\right) -\left( |G|^{-1}\sum _{g\in G\setminus K}\chi (g)^l\right) \\= &   |G|^{-1}\sum _{g\in G}\phi (g)\overline{\chi (g)^l}=\langle \phi ,\chi ^l\rangle , \end{aligned}$$

as desired. $$\square $$

By Lemma 2.1, to prove Theorem 1.1, it only remains to show that $o_K(l)-o(l)$ is the number of regular *G*-orbits on $\Omega ^l.$

### Lemma 2.2

Fix $l\in {\mathbb {N}}.$ Then $o_K(l)-o(l)$ is the number of regular *G*-orbits on $\Omega ^l.$

### Proof

Since $|G:K|=2,$ each orbit of *G* on $\Omega ^l$ is the union of either one or two *K*-orbits. Thus, $o_K(l)-o(l)$ is the number of *G*-orbits which are the unions of exactly two *K*-orbits. Since every *K*-orbit has fewer than |*G*| points, it suffices to show that any *G*-orbit which is the union of two *K*-orbits is regular.

Let ${\mathcal {A}}\in \Omega ^l$ be such that ${\mathcal {A}}^G:=\{{\mathcal {A}}^g: g\in G\}$ is the union of two *K*-orbits. Then without loss of generality $2|{\mathcal {A}}^K|\le |{\mathcal {A}}^G|,$ thus from the orbit-stabiliser lemma and the fact that $|G|=2|K|,$ we deduce that $|G_{\mathcal {A}}|\le |K_{\mathcal {A}}|.$ But $K_{\mathcal {A}}\le G_{\mathcal {A}},$ whence $G_{{\mathcal {A}}}=K_{{\mathcal {A}}}\le K.$ Since $\phi$ is base-controlling, it follows that ${\mathcal {A}}$ is a base for *G*. That is, ${\mathcal {A}}^G$ is a regular *G*-orbit, as was to be shown. $\square$

Putting together Lemmas 2.1 and 2.2, we deduce Theorem 1.1.

## Examples and applications

In this section, we present some examples and non-examples of groups admitting base-controlling homomorphisms, and we give an application of Theorem 1.3.

We begin by noting that the character ${\textrm{sgn}}$ of $\textrm{S}_{n,k}$ is base-controlling. Indeed, the pointwise stabiliser of any collection of *k*-subsets is a direct product of symmetric groups—the only symmetric group containing no odd permutations is the trivial group. Thus, we deduce from Theorem 1.2 the formula of Mecenero and Spiga.

### Theorem 3.1

([4, Theorem 1.1]). Let $n>2k$ be positive integers. Then $b(S_{n,k})$ is the minimum *l* such that

$$\begin{aligned} \sum _{\begin{array}{c} \pi \vdash n\\ \pi =(1^{c_1},2^{c_2},\ldots ,n^{c_n}) \end{array}}(-1)^{n-\sum _{i=1}^nc_i}\frac{n!}{\prod _{i=1}^ni^{c_i}c_i!}\left( \sum _{\begin{array}{c} \eta \vdash k\\ \eta =(1^{b_1},2^{b_2},\ldots ,k^{b_k}) \end{array}}\prod _{j=1}^k{c_j\atopwithdelims ()b_j}\right) ^l&\ne 0. \end{aligned}$$

### Proof

Since ${\textrm{sgn}}$ is base-controlling, $b(\textrm{S}_{n,k})$ is the minimum *l* such that $\langle {\textrm{sgn}},\chi ^l\rangle \ne 0.$ Let $C_1,C_2,\ldots , C_m$ be the conjugacy classes of $\textrm{S}_{n,k},$ and let $g_i$ be a representative of the class $C_i$ for each *i*. Then

$$\begin{aligned} \langle {\textrm{sgn}},\chi ^l\rangle =n!^{-1}\sum _{i=1}^m{\textrm{sgn}}(g_i)|C_i|\chi (g_i)^l. \end{aligned}$$

Now, recall that the conjugacy classes of $\textrm{S}_{n,k}$ are in correspondence with the partitions of *n*—it is easy to see that the sign of a permutation *g* corresponding to the partition $\pi =(1^{c_1},2^{c_2},\ldots ,n^{c_n})$ is exactly $(-1)^{n-\sum _{i=1}^nc_i}.$ Moreover, it is well-known that the size of the conjugacy class of such an element is precisely $\frac{n!}{\prod _{i=1}^ni^{c_i}c_i!}.$ Similarly, one can calculate that

$$\begin{aligned} \chi (g)=\sum _{\begin{array}{c} \eta \vdash k\\ \eta =(1^{b_1},2^{b_2},\ldots ,k^{b_k}) \end{array}}\prod _{j=1}^k{c_j\atopwithdelims ()b_j}, \end{aligned}$$

hence the result. $\square$

We now point out that admitting a base-controlling homomorphism is a property of a permutation group, not of an abstract group. Indeed, as observed above, ${\textrm{sgn}}$ is a base-controlling homomorphism for $\textrm{S}_{n,k},$ however, it is not the case that ${\textrm{sgn}}$ is always base-controlling for $\textrm{S}_n.$ Take for example $\textrm{S}_{15}$ acting on the partitions of [15] into exactly 3 parts of size 5,  with permutation character $\chi .$ By  [5, Theorem 1.1], this group has base size 3,  however one can compute using GAP [6] that $\langle {\textrm{sgn}},\chi ^2\rangle \ne 0.$

Our final application is towards determining the base size of large base groups. Given integers *n* and *k*,  define $\textrm{A}_{n,k}$ to be the alternating group acting on the *k*-subsets of [*n*]. A permutation group *G* is *large base* if there exist integers *m*,  *r*,  and *k* such that $\textrm{A}_{m,k}^r \unlhd G \le \textrm{S}_{m,k} \wr \textrm{S}_r,$ where the wreath product acts with product action.

### Proposition 3.2

Let *m*,  *r*,  and *k* be integers,  and let $\textrm{A}_{m,k}^r \unlhd G \le \textrm{S}_{m,k} \wr \textrm{S}_r.$ Let $\chi _{m-1}$ and $\chi _m$ be the permutation characters of $\textrm{S}_{m-1,k}$ and $\textrm{S}_{m,k},$ respectively. Then

$$\begin{aligned} \min \{l \in {\mathbb {N}}: \langle {\textrm{sgn}},\chi _{m-1}^l\rangle _{\textrm{S}_{m-1}}>0\}\le b(G)\le \min \{l \in {\mathbb {N}}: \langle {\textrm{sgn}},\chi _m^l\rangle _{\textrm{S}_m}\ge r\}. \end{aligned}$$

### Proof

We show that $b(\textrm{A}_{m,k}^r)=\min \{l \in {\mathbb {N}}: \langle {\textrm{sgn}},\chi _{m-1}^l\rangle _{\textrm{S}_{m-1}}>0\},$ and that $b(\textrm{S}_{m,k} \wr \textrm{S}_r)=\min \{l \in {\mathbb {N}}: \langle {\textrm{sgn}},\chi _m^l\rangle _{\textrm{S}_{m}}\ge r\},$ from which the result will follow.

Consider first the lower bound. Note that $b(\textrm{A}_{m,k}^r)=b(\textrm{A}_{m,k})=b(\textrm{S}_{m-1,k})$ by [3, Theorem 1.1], hence the lower bound holds by Theorem 1.2. That

$$\begin{aligned} b(\text {S}_{m,k} \wr \text {S}_r)=\min \{l \in {\mathbb {N}}: \langle {\text {sgn}},\chi _m^l\rangle _{\text {S}_{m}}\ge r\} \end{aligned}$$

follows immediately from Theorem 1.3 and the fact that $D(\textrm{S}_r)=r.$ $\square$

### Remark

Of course, if more is known about the structure of *G*,  then one may apply Theorem 1.3 directly to obtain a more precise formula for *b*(*G*).

So far the only example of a group which admits a base-controlling homomorphism that we have seen is the symmetric group—we now provide an alternative example.

Let $G={\textrm{PSL}}_2(7):2$ acting sharply 3-transitively with point stabiliser 7 : 6,  and hence 2-point stabiliser 6. Consider the map $\phi : G\rightarrow \{1,-1\}$ with kernel ${\textrm{PSL}}_2(7).$ The map $\phi$ is base-controlling: if ${\mathcal {A}}$ is such that $\phi (G_{\mathcal {A}})=1,$ then since $\phi (7:6)=\phi (6)=\{1,-1\},$ it follows that ${\mathcal {A}}$ contains at least three distinct points. Since *G* is sharply 3-transitive, any triple of distinct points forms a base for *G*,  that is, $G_{{\mathcal {A}}}=1$ as desired. Of course, the above argument hints at a construction of many groups with a similar structure.

We conclude the paper with a very nice question asked by an anonymous referee: is it true that for every permutation group *G* with permutation character $\chi ,$ there exists an irreducible character $\phi$ such that $b(G)=\min \{l\in {\mathbb {N}}: \langle \phi , \chi ^l\rangle \ne 0\}?$ The answer turns out to be ‘no’. Indeed, let *a*, *b*, *c* be distinct positive integers and let *G* be the Klein 4-group $\{1,-1\}\times \{1,-1\}$ acting on $\{a,-a,b,-b,c,-c\}$ via $a^{(x,y)}=xa,$ $b^{(x,y)}=yb,$ and $c^{(x,y)}=xyc.$ The permutation character $\chi$ of this action then contains all four irreducible characters of *G*,  and so $\min \{l\in {\mathbb {N}}: \langle \phi , \chi ^l\rangle \ne 0\}=1$ for all $\phi$ irreducible, whereas $b(G)=2.$
